# Novel CRISPR-Associated Gene-Editing Systems Discovered in Metagenomic Samples Enable Efficient and Specific Genome Engineering

**DOI:** 10.1089/crispr.2022.0089

**Published:** 2023-06-01

**Authors:** Rebecca C. Lamothe, Meghan D. Storlie, Diego A. Espinosa, Rachel Rudlaff, Patrick Browne, Jason Liu, Andres Rivas, Audra Devoto, Jennifer Oki, Ashcon Khoubyari, Daniela S. Aliaga Goltsman, Jyun-Liang Lin, Cristina N. Butterfield, Christopher T. Brown, Brian C. Thomas, Gregory J. Cost

**Affiliations:** Metagenomi, Inc., Emeryville, California, USA.

## Abstract

Development of medicines using gene editing has been hampered by enzymological and immunological impediments. We described previously the discovery and characterization of improved, novel gene-editing systems from metagenomic data. In this study, we substantially advance this work with three such gene-editing systems, demonstrating their utility for cell therapy development. All three systems are capable of reproducible, high-frequency gene editing in primary immune cells. In human T cells, disruption of the T cell receptor (TCR) alpha-chain was induced in >95% of cells, both paralogs of the TCR beta-chain in >90% of cells, and >90% knockout of β2-microglobulin, *TIGIT*, *FAS*, and *PDCD1*. Simultaneous double knockout of *TRAC* and *TRBC* was obtained at a frequency equal to that of the single edits. Gene editing with our systems had minimal effect on T cell viability. Furthermore, we integrate a chimeric antigen receptor (CAR) construct into *TRAC* (up to ∼60% of T cells), and demonstrate CAR expression and cytotoxicity. We next applied our novel gene-editing tools to natural killer (NK) cells, B cells, hematopoietic stem cells, and induced pluripotent stem cells, generating similarly efficient cell-engineering outcomes including the creation of active CAR-NK cells. Interrogation of our gene-editing systems' specificity reveals a profile comparable with or better than Cas9. Finally, our nucleases lack preexisting humoral and T cell–based immunity, consistent with their sourcing from nonhuman pathogens. In all, we show these new gene-editing systems have the activity, specificity, and translatability necessary for use in cell therapy development.

## Introduction

In the years since its discovery, CRISPR-associated gene-editing systems have been exploited to revolutionize how diseases are identified, diagnosed, and treated across a range of therapeutic areas.^1–6^ In brief, CRISPR-based gene-editing reagents are generally used as two-component systems: a nuclease protein is complexed with a single-guide RNA (sgRNA) designed to target a specific site in the genome.^1,7,8^ Much work has gone into engineering both nucleases and guides to optimize function and stability across an array of cell types.^1,9^ The CRISPR-associated nucleases most commonly used for double-stranded DNA targeting belong to class II and can be binned into two groups, type II and type V, that differ in the position of their protospacer-adjacent motif (PAM) vis-à-vis the spacer region (3′ vs. 5′) and the DNA ends that result from the cleavage reaction (blunt vs. overhung).^10–12^

Although *Streptococcus pyogenes* Cas9 is a highly active gene-editing enzyme, its use is complicated by a low editing specificity. Conversely, Cas12a, a more accurate type V enzyme,^13–15^ remains underutilized owing to its generally lower activity relative to Cas9. Furthermore, Cas9 is derived from a *Streptococcus* bacterium, a very commonly pathogenic genus. As a result, between one-third and half of people have a preexisting immune response to the Cas9 enzyme^16^ and thus are less-than-optimal candidates for a Cas9-based gene-editing therapy. Despite immense recent progress, development of gene editing–based medicines nonetheless has been hindered by these limitations of activity, specificity, and immunogenicity.

To ameliorate these issues and expand the repertoire of available gene-editing tools, we established a metagenomics pipeline to discover new type II and type V effectors. This work led to the discovery of ∼5000 CRISPR loci.^16–18^ We characterized 136 of these type II systems, showed positive activity for 96/136 *in vitro*, and demonstrated activity of 14/21 in immortalized mammalian cells. Before this, we characterized six type V systems *in vitro* and demonstrated that four, including our lead type V enzyme, MG29-1, were active in immortalized mammalian cells.

Here we expand this work substantially, generating proof-of-concept gene-editing outcomes in a wide variety of primary cells with our lead type II and type V enzymes. We also demonstrated the activity of MG3-6/3-4, a chimeric type II enzyme generated by swapping in the C-terminal portion of MG3-4 (comprising the RuvC-III, WED, and PI domains) into the N-terminal sequence of MG3-6 using a conserved two-amino acid site in the C terminus as the breakpoint.^18,19^

Gene editing has been applied to cell therapy with many types of primary immune cells—especially T cells—via electroporation of Cas9 ribonucleoprotein particles (RNPs). Such engineering has been used to knockout the T cell receptor (TCR), checkpoint inhibitors, and to knock-in chimeric antigen receptors (CARs), among many examples.^15,20–24^ Here, we use three of our new gene-editing systems (two type II and one type V) to create gene-edited T cells, B cells, natural killer (NK) cells, induced pluripotent stem cells (iPSCs), and hematopoietic stem cells (HSCs), including proof-of-concept T cell and NK cell therapies.

We edit these cells at more than a dozen therapeutically relevant loci, performing both gene knockout and transgene knock-in and creating, among others, cells with multiplexed gene-editing outcomes that will enable both autologous TCR-based T cell therapy and allogeneic CAR-T cell therapy. We find that our editing reagents have specificity equal to or better than Cas9 in both immortal and primary cells but with essentially no preexisting immunoreactivity. With a high degree of activity, specificity, and translatability, these new tools will make possible a great diversity of gene editing–based therapies.

## Materials and Methods

### Protein purification

Auto-inducing media was inoculated with fresh plate scraped transformants in BL21 (DE3), and the cultures were incubated at 37°C for 3 h then cooled to 18°C and shaken overnight. After the first night, the media were supplemented with IPTG (1 mM) and grown for 48 hours. After cell harvest, cells were resuspended in lysis buffer (50 mM Na_2_HPO_4_ pH 8, 800 mM NaCl, 10 mM imidazole, BugBuster, Benzonase) and lysed via sonication. The lysate was purified on a HisTrap HP column eluting with a linear gradient from 100 to 500 mM imidazole in 50 mM Na_2_HPO_4_ pH 8 and 100 mM NaCl. Fractions were pooled and diluted to 10 mL, then supplemented with 0.4 mg TEV protease, 1 mM DTT, and 1 mM EDTA and incubated for 48 h at 4°C.

The TEV cleaved product was loaded onto HiTrap SP HP for ion exchange and eluted with a 100–1000 mM KCl gradient with 20 mM HEPES pH 7. Relevant fractions were pooled and buffer exchanged using PD-10 columns according to the manufacturer's instructions into storage buffer 40 mM HEPES pH 7.0, 400 mM KCl. After addition of 80% glycerol and DTT the storage conditions were 20 mM HEPES pH 7.0, 200 mM KCl, 1 mM DTT, and 40% glycerol pH.

### Flow cytometry

For surface and intracellular staining, 100,000 cells per sample were harvested and stained with the LIVE/DEAD™ Fixable Near IR (780) Viability Kit (ThermoFisher No. L34992) according to the manufacturer's protocol for 10 min at room temperature. Cells were subsequently washed with fluorescence-activated cell sorting (FACs) buffer (1 × phosphate-buffered saline [PBS] supplemented with 5% fetal bovine serum [FBS]) and stained for surface markers for 30 min at 4°C. For direct staining of the B cell maturation antigen (BCMA)-CAR, biotinylated human BCMA/TNFRSF17 protein, Fc and Avi Tag was purchased from Acro Biosystems (No. BC7-H82F0). After viability staining, cells were resuspended in 100 μL FACs buffer containing 3 μg/mL biotinylated human BCMA protein and incubated at 4°C for 1 h. Cells were washed with FACs buffer and then resuspended in FACs buffer containing 1:400 dilution of phycoerythrin (PE)-streptavidin (Biolegend No. 405203) and other monoclonal antibodies of interest and incubated at 4°C for 1 h.

Cells were washed twice with FACs buffer before data acquisition using an Attune NxT Flow Cytometer (ThermoFisher). For staining of intracellular markers, the Inside Stain kit (Miltenyi No. 130-090-477) was used according to the manufacturer's instructions. The following monoclonal antibodies were purchased from Biolegend: APC anti-TCR α/β (clone IP26) and PE anti-BCMA (clone 19F2); from Miltenyi: VioBlue anti-CD271 (LNGFR) (REA844), APC anti-Oct3/4 Isoform A (REA338), and FITC anti-Sox2 (REA320); and from ThermoFisher: APC anti-CD34 (clone 4H11), APC anti-CD56 (clone CMSSB), and APC anti-CD19 (clone HIB19). Fluorescence-minus-one controls were used for gating.

### RNP formation, nucleofection, and indel analysis

RNPs were formed by complexing either 104 pmol MG3-6 (or MG3-6/4) and 120 pmol sgRNA or 120 pmol MG29-1 and 250 pmol crRNA at room temperature for 30 min. RNPs were nucleofected using the protocols described hereunder. Cells were harvested 72 h postelectroporation for genomic DNA extraction using QuickExtract (Lucigen No. 09050) and processed for next-generation sequencing on an Illumina Miseq. Resulting data were analyzed with an in-house indel calculator script.

### Primary human T cell culture and nucleofection

Cryopreserved human peripheral blood mononuclear cells (PBMCs) were purchased from StemCell Technologies (StemCell No. 70025) and thawed per the manufacturer's instructions. T cells were purified using the EasySep™ Human T Cell Isolation Kit from StemCell Technologies (StemCell No. 17951) and activated using a T Cell Activation/Expansion Kit sourced from Miltenyi Biotec (Miltenyi No. 130-091-441) according to the manufacturer's protocol. T cells were activated and cultured at 500,000 cells/mL in Immunocult-XF T Cell Expansion Medium (StemCell No. 10981) supplemented with 5% CTS Immune Cell SR (Gibco No. A2596101) and 50 ng/mL rhIL-2 (Peprotech No. 200-02) for 72 h, after which the magnetic activation beads were removed. Cells were resuspended in fresh culture medium at 1 million cells per milliliter and expanded in culture for an additional 24 h before gene-editing experiments.

For gene-editing experiments, RNPs were formed as described previously and electroporated into 200,000 T cells using the P3 Primary Cell kit (Lonza No. V4XP-3032) in the Lonza 4D nucleofector using the EO-115 program. After electroporation, T cells were resuspended in 200 μL of culture medium supplemented with 50 ng/mL rhIL-2 and cultured in 96-well plate format. For experiments using adeno-associated virus (AAV)-6 transduction, the relevant donor AAV-6 at the indicated multiplicity of infection (MOI) was diluted in culture medium before electroporation, and added to cells immediately after the electroporation event. Seventy-two hours postelectroporation, cells were harvested for flow cytometry and genomic DNA extraction.

### Primary human NK cell culture and nucleofection

Cryopreserved human peripheral blood CD56^+^ cells were purchased from StemCell Technologies (StemCell No. 70037) and expanded using the Cloudz Human NK Cell Expansion kit (R&D Systems No. CLD004) according to the manufacturer's protocol. Upon thaw, NK cells were seeded in T25 flasks with Cloudz anti-CD2- and anti-NKp46-coated microspheres and cultured in ExCellerate™ human NK cell expansion media (R&D Systems No. CCM032) supplemented with 10% FBS and varying concentrations of rhIL-2, rhIL-12, rhIL-18, and rhIL-21 (R&D Systems Nos. 202-IL, 219-IL, 9124-IL, 8879-IL). On day 10 of culture, Cloudz microspheres were dissolved using the provided release buffer before gene-editing experiments. For gene-editing experiments, RNPs were formed as described previously and electroporated into 500,000 NK cells in a custom buffer composed of 5 mM KCl, 15 mM MgCl_2_, 15 mM HEPES, 150 mM Na_2_HPO_4_/NaH_2_PO_4_ (phosphate buffer), and 50 mM mannitol at pH 7.2 in the Lonza 4D nucleofector using the CM-137 program.

Following electroporation, NK cells were resuspended in 500 μL NK cell expansion media supplemented with rhIL-2, rhIL-12, rhIL-18, and rhIL-21 and cultured in 24-well plate format. For experiments using AAV-6 transduction, the relevant donor AAV-6 at the indicated MOI was diluted in culture media before electroporation, and added to cells immediately following the electroporation event. Seventy-two hours postelectroporation, cells were harvested for flow cytometry and genomic DNA extraction as described previously.

### Primary human B cell culture and nucleofection

Cryopreserved human peripheral blood B cells were purchased from StemCell Technologies (StemCell No. 70023) and expanded using the ImmunoCult™ Human B Cell Expansion Kit according to the manufacturer's protocol for 48 h before nucleofection. For gene-editing experiments, RNPs were formed as described previously and electroporated into 200,000 B cells using the P3 Primary Cell kit (Lonza No. V4XP-3032) in the Lonza 4D nucleofector using the EO-117 program. Following electroporation, B cells were resuspended in 1 mL of culture medium and cultured in 24-well plate format. For experiments using AAV-6 transduction, the relevant donor AAV-6 at the indicated MOI was diluted in culture media before electroporation and added to cells immediately following the electroporation event. Seventy-two hours postelectroporation, cells were harvested for flow cytometry and genomic DNA extraction as described previously.

### Primary HSC culture and nucleofection

Mobilized peripheral blood CD34^+^ cells were acquired from AllCells and cultured in StemCell StemSpan™ SFEM II media (StemCell No. 09605) supplemented with StemSpan CC110 (StemCell No. 02697) cytokine cocktail for 48 h before nucleofection. For gene-editing experiments, RNPs were formed as described previously and electroporated into 200,000 HSCs using the P3 Primary Cell kit (Lonza No. V4XP-3032) in the Lonza 4D nucleofector using the CA-137 program. Following electroporation, HSCs were resuspended in 1 mL of culture medium and cultured in 24-well plate format. For experiments using AAV-6 transduction, the relevant donor AAV-6 at the indicated MOI was diluted in culture media before electroporation, and added to cells immediately after the electroporation event. Seventy-two hours postelectroporation, cells were harvested for flow cytometry and genomic DNA extraction as described previously.

### iPSC culture and nucleofection

ATCC-BXS0116 human iPSCs (ATCC No. ACS-1030) were cultured on Corning Matrigel-coated plasticware in mTESR Plus media (StemCell No. 100-0276) containing 10 μM ROCK inhibitor Y-27632 (StemCell No. 72302) for 24 h before nucleofection. For gene-editing experiments, RNPs were formed as described previously and electroporated into 200,000 iPSCs using the P3 Primary Cell kit (Lonza No. V4XP-3032) in the Lonza 4D nucleofector using the CA-137 program. Immediately before electroporation iPSCs were harvested and dissociated into single cells using Accutase (StemCell No. 07922) according to the manufacturer's protocol. Following electroporation, iPSCs were resuspended in 1 mL of culture medium supplemented with 10 μM ROCK inhibitor Y-27632 and cultured in 24-well plate format for an additional 24 h.

For experiments using AAV-6 transduction, the relevant donor AAV-6 at the indicated MOI was diluted in culture media before electroporation, and added to cells immediately following the electroporation event. Rock inhibitor was removed 24 h postelectroporation. Seven days postelectroporation, cells were harvested for flow cytometry and genomic DNA extraction using Accutase. All primary cells were purchased from commercial organizations with in-house institutional review boards and other safeguards in-place to ensure the ethical sourcing of the material.

### Cytotoxicity assay and interferon gamma enzyme-linked immunosorbent assay

Effector cells transduced to express the BCMA-CAR construct were labeled with CFSE by staining with CellTrace™ CFSE Cell Proliferation Kit (ThermoFisher No. C34554) according to the manufacturer's protocol. Target cells (either K562 parental cells or isogenic K562 cells engineered to express BCMA) were labeled with CellTrace Violet Cell Proliferation Kit (ThermoFisher No. C34571) according to the manufacturer's protocol. Labeled effector cells normalized for CAR expression were plated in coculture with target cells at various effector-to-target ratios in a 96-well U-bottom plate and placed in a 37°C 5% CO_2_ incubator. After 24 or 48 h, cells were spun down and cell culture supernatant harvested for measuring secreted interferon gamma (IFN-γ). Cells were stained with the LIVE/DEAD Fixable Near IR (780) Viability Kit (ThermoFisher No. L34992) according to the manufacturer's protocol and samples were acquired on an Attune NxT Flow Cytometer.

Data were analyzed in FlowJo software V10.7.1 and specific lysis calculated using the following equation: Specific Lysis = 1 − [(Number of live target cells in a given effector:target sample)/(Number of live target cells in target-only well)]. For analysis of secreted IFN-γ, cell culture supernatants were diluted 1:200 in calibrator diluent provided in the human IFN-γ Quantikine enzyme-linked immunosorbent assay (ELISA) Kit (R&D Systems No. DIF50) and the assay performed according to the manufacturer's protocol.

### Lenti library construction for in cell PAM determination assay

Oligo pools comprising 16,384 ssDNA designed with unique 18 nt barcodes and 4 target sequences adjacent to 2 combinatorial “T-(7N)-A” PAM libraries were synthesized by Twist. Oligos were made double stranded, and cloned into a Lentiviral transfer plasmid using HiFi Assembly (NEB) alongside a negative assembly reaction without the dsDNA insert library. Assembly reactions were transformed into electrocompetent Endura Duo *Escherichia coli* cells (Endura), and were recovered at 30°C for 1 h. Ten microliters of the recovery culture, representing 1% of the pool, were plated on lysogeny broth (LB)-ampicillin agar plates and the rest of the recovery culture was grown for 16 h at 30°C in 500 mL of LB-ampicillin medium. Colony-forming units from plated cultures were counted and noninsert cultures were subtracted as background. Insert-containing transformations were then extrapolated for library diversity, and the process was continued until 10 × coverage of the original oligo pool was achieved.

Lentiviral transfer plasmids were then isolated from overnight liquid cultures using a Megaprep kit (Qiagen) and made endonuclease free using an Endofree column (Zymo). Transfection of 2 million 293T cells was performed with 45 μL LT1 transfection reagent (Mirus Bio), and 8 μg psPAX2, 1 μg pMD2.G, and 9 μg lentiviral library transfer plasmid. Supernatant of viral production cultures were harvested after 72 h at 37°C was filter cleared with a 0.45 μm PES filter (Millipore) and 5 mL of supernatant was transferred to each 2.5 million cells of 293T and K562 cultures for transduction for 72 h. Transduced cultures were selected with 2 μg/mL puromycin at 300,000 cells/mL for 4 days with cell dilution and addition of puromycin every 2 days.

### Transfection, processing, and analysis of lentiviral library cell lines

Three hundred ten picomoles effector and 500 picomoles sgRNA were used to transfect 2 million cells using a 4D-Nucleofector X Kit (Lonza). Cells were recovered for 72 h at 37°C and genomic DNA was isolated using the NucleoSpin Blood L Midi Kit (Macharey Nagel). Nextera compatible primers with diversity stub sequences ranging between 1 and 5 nt were pooled to 500 pM to amplify 90 ng/μL gDNA in NEBnext Q5 Master mix (NEB) over 23 amplification cycles. Ten 100 μL polymerase chain reaction (PCR) reactions of each isolated gDNA condition were pooled and processed with 1 × SPRI beads (manufacturer) and sequenced using a 150 cycle MiSeq kit (Illumina). Sequences were processed using CRISPResso2, and indel profiles were visualized using SeqLogo.

### Double-strand break detection assay

HEK293T cells were nucleofected with precomplexed RNP consisting of either 12 pmol Cas9 and 60 pmol sgRNA, 104 pmol MG3-6 and 120 pmol sgRNA, or 120 pmol MG29-1 and 120 pmol sgRNA, using the Lonza 4D electroporation system. In parallel, cells were cotransfected with 50 pmol annealed dsODN. After 72 h, cells were trypsinized and gDNA extracted using the Kingfisher MagMAX DNA Multi-Sample Ultra 2.0 Kit (Catalog No. A36570). Four hundred nanograms of high molecular weight gDNA was fragmented, end-repaired, and ligated using the NEB FS DNA Library Prep Kit (Catalog No. E7805). Fragments between 350 and 600 bp in length were amplified to enrich for dsODN-proximal regions using dsODN-specific primers in both the positive and negative orientations. Resulting libraries were amplified for next generation sequencing (NGS) on Illumina Miseq.

### Detection and quantification of 53BP-1 puncta

HEK293T cells from ATCC (CRL-3216) were cultured according to ATCC protocols. To measure cellular responses to RNP transfection, RNPs were formed as described previously and transfected into 2E5 HEK293T cells using the SF cell line kit (Lonza No. V4XC-2032) and a Lonza 4D nucleofector on the HEK293 protocol (CM-130). Transfected cells for each RNP were split for the number of conditions needed and plated into 24-well plates containing 12 mm poly-l-lysine–coated coverslips for immunofluorescence analysis or wells without coverslips for NGS analysis. Coverslips were processed for immunofluorescence 6 and 24 h post-transfection.

For immunofluorescence assay, coverslips were washed with ice-cold PBS, cells were fixed in ice-cold 4% paraformaldehyde in PBS for 15 min, permeabilized with 0.1% Triton-PBS for 10 min, blocked in 3% bovine serum albumin (BSA)-PBS for 1 h at room temperature or overnight at 4°C, incubated with primary antibody in 3% BSA-PBS for 1 h at room temperature, incubated with secondary antibody in 0.5% BSA-PBS for 45 min at room temperature, and mounted on slides with ProLong Diamond with 4′,6-diamidino-2-phenylindole (DAPI; ThermoFisher No. P36932). Antibodies used were rabbit α-53BP1 (Bethyl Laboratories No. A300-273A) at 1:2500 and goat α-rabbit cross-adsorbed Alexa-594 secondary (ThermoFisher No. A-11012). Cells were imaged on an EVOS M5000 microscope with the DAPI and TexasRed filter cubes at 100 × and acquired as 16-bit TIFF files.

Images were analyzed in Fiji as follows: for each field of view, region of interest (ROI) were set on the DAPI image using the Threshold function to define nuclear regions followed by the Analyze Particles function to set ROI. To identify foci, the Find Maxima function was used on the corresponding image from the TRITC channel. Prominence was determined for each experiment based on the number at which foci were robustly detected across control and experimental conditions and was kept the same across all conditions for each experiment. Foci were output as single points, the ROI masks from the DAPI image applied to the foci output, and the number of foci per nucleus was measured. Foci were measured for >40 cells per condition. For NGS, cells were collected 54–72 h post-transfection and gDNA was obtained using a Qiagen DNeasy Blood and Tissue Kit (No. 69504). Amplicons encompassing the target cut site were amplified using appropriate primers and were prepared for next-generation sequencing as described.

### Nuclease production for preexisting immunity assay

MG29-1 and MG3-6 were expressed in and purified from human HEK293 cells using the Expi293™ Expression System Kit (ThermoFisher Scientific). In brief, 293 cells were lipofected with plasmids encoding the nucleases driven by a strong viral promoter. Cells were grown in suspension culture with agitation and harvested 2 days post-transfection. The nuclease proteins were fused to a Six-His affinity tag and were purified by metal-affinity chromatography to 50–60% purity. Parallel lysates were made from mock-transfected cells and were subjected to an identical metal-affinity chromatography process. Cas9 was purchased from IDT and is >95% pure.

### ELISA assay

MaxiSorp^®^ ELISA plates (Thermo Scientific) were coated with 0.5 μg of nucleases or control proteins diluted in 1 × PBS and incubated overnight at room temperature. Plates were then washed and incubated with a 1% (w/v) BSA (Sigma-Aldrich)/1 × PBS solution (1% BSA-PBS) for an hour at room temperature. After another washing step, wells were incubated in duplicate for 1 h at room temperature with 48 separate serum samples (BioChemed) taken from randomly selected donors (1:50 dilution in 1% BSA-PBS). Plates were then washed and incubated for an hour at room temperature with a peroxidase-labeled goat anti-human (Fcγ fragment-specific) secondary antibody (Jackson Immuno Research), diluted 1:50,000 in 1% BSA-PBS. The assay was developed using a 3,3′,5,5′-tetramethylbenzidine Liquid Substrate System kit (Sigma-Aldrich), according to the manufacturer's specifications. Antibody titers were reported as average absorbance values measured at 450 nm. Tetanus toxoid was purchased from Sigma-Aldrich.

### Intracellular cytokine staining

Cryopreserved PBMCs from healthy donors were purchased from StemCell Technologies, Inc. Cells were thawed at 37°C and resuspended in 8 mL of complete Roswell Park Memorial Institute (RPMI) 1640 medium with 5% human AB serum, 1000 U/mL penicillin, and 1000 μg/mL streptomycin. Cells were spun at 400 *g* for 10 min, the medium was aspirated, and cells were then resuspended in 2 mL of complete RPMI medium; 9E5 PBMCs were plated at a concentration of 4.5E6 cells per milliliter in 96-well V-bottom plates. Plated cells were rested overnight, and each antigen was added at a concentration of 10 μg/mL. Cells were incubated for 2 h with antigen and then brefeldin A (BD Biosciences) was added at a concentration of 10 μg/mL and cells were incubated for another 4 h. PBMCs were washed once with PBS and stained for viability using LIVE/DEAD Fixable Aqua Dead Cell Stain Kit (ThermoFisher).

Cells were then washed twice in staining buffer (5% FBS and 0.01% sodium azide in PBS) and surface stained for CD3 (Brilliant Violet 421 anti-human CD3 antibody, clone UCHT1; Biolegend), CD4 (Brilliant Violet 650 anti-human CD4 antibody, clone OKT4; Biolegend), and CD8a (Brilliant Violet 785 anti-human CD8 antibody, clone SK1; Biolegend). Cells were subsequently fixed and permeabilized using a Cytofix/Cyptoperm kit (BD Biosciences), and stained for intracellular cytokines including IFN (APC anti-human IFN antibody, clone 4S.B3; Biolegend), TNF (FITC anti-human TNF antibody, clone MAb11; Biolegend), and IL-2 (PerCP/Cyanine5.5 anti-human IL-2 antibody, clone MQ1-17H12; Biolegend). Finally, PBMCs were washed twice and resuspended in a permeabilization buffer (BD Biosciences) and analyzed with an Attune NxT flow cytometer (ThermoFisher).

## Results

### Novel type II and type V nucleases can be used to engineer primary T cells into highly active, TCR-deficient CAR-T cells

**FIG. 1.**
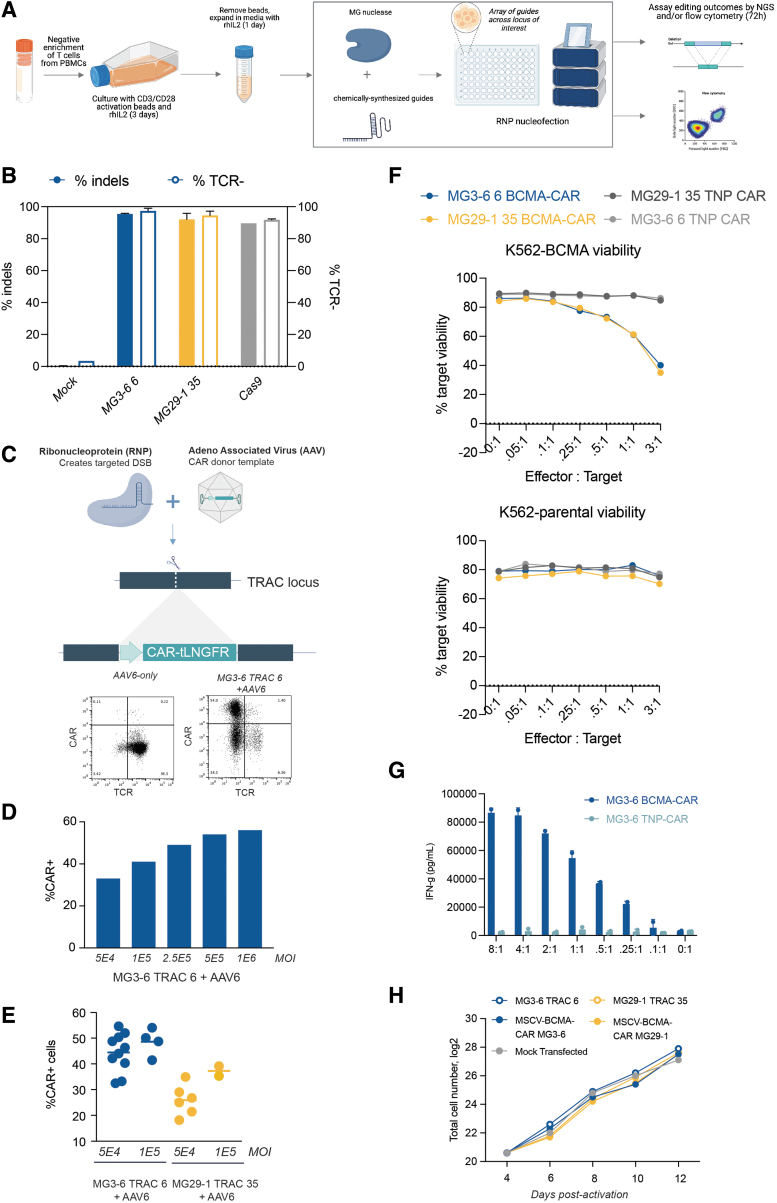
Engineering of primary T cells using novel type II and type V nucleases. **(A)** Schematic of T cell purification, stimulation, electroporation, and recovery workflow; **(B)** TCR knockout of top MG3-6, MG29-1, and Cas9 guides assayed NGS (filled bar) and flow cytometry (open bar) in parallel across three biological replicates. **(C)** Schematic of CAR-T cell creation and exemplary flow cytometry plot assaying CAR expression. CAR integration is into *TRAC* exon 1 for MG29-1 and into exon 3 for MG3-6. Cells were recovered post-transfection in medium containing AAV and assayed for CAR expression (*y* axis) by binding of fluorescently labeled BCMA and for TCR expression (*x* axis); **(D)** dose response of CAR integration and expression in T cells as a function of AAV genomes transduced; **(E)** CAR integration into T cells from 10 T cell donors with MG3-6 and MG29-1 and either 5E4 or 1E5 MOI; **(F)** dose-dependent, CAR- and antigen-specific cytotoxic activity of MG3-6- and MG29-1-based CAR-T cells when exposed to cells expressing the CAR target antigen BCMA. Data from the MG3-6 anti-BCMA CAR are given in blue, from the MG29-1 anti-BCMA CAR are given in yellow, and an anti-TNP CAR integrated with MG3-6 or MG29-1 in gray; cytotoxic response to parental line is shown in bottom panel; **(G)** induction of IFN-γ production during exposure to BCMA-expressing cell line. Levels of IFN-γ in T cells expressing either the BCMA-CAR-T (in blue) or control TNP-CAR (in gray); **(H)** expansion of edited and unedited T cells with or without gene editing. Mock transfected cells are indicated in gray circles, cells transfected only with MG3-6 are indicated with open blue circles, MG3-6-derived CAR-Ts with solid blue circles, MG29-1 with yellow circles, and MG29-1-derived CAR-Ts with dark yellow circles. AAV, adeno-associated virus; BCMA, B cell maturation antigen; CAR, chimeric antigen receptor; IFN-γ, interferon gamma; MOI, multiplicity of infection; NGS, next generation sequencing; TCR, T cell receptor.

Allogeneic T cell therapy is a promising therapeutic mode but it is complicated by the near certainty of graft-versus-host-disease if appreciable allo-reactive T cells are present in the drug product. Elimination of the endogenous TCR has been used to ablate alloreactivity.^25,26^ We thus endeavored to inactivate the alpha chain of the TCR (*TRAC*) by targeting the constant region of the gene with our new gene-editing systems. To that end, we purified T cells from cryopreserved PBMCs and activated them with CD3, CD28, and rhIL-2 (Fig. 1A). We designed a panel of guide RNAs tiling across all three *TRAC* exons with our flagship type II (MG3-6) and type V (MG29-1) nucleases, then made and electroporated RNPs into T cells.

We assessed knockout efficiency 3 days post-transfection by next-generation DNA sequencing and by flow cytometry for the presence of the TCR. Strikingly, we were able to knockout *TRAC* as well or better than a highly active Cas9 sgRNA, routinely achieving 90–95% *TRAC* editing (Fig. 1B). We observed a high correlation between indel generation and functional inactivation by flow cytometry (data not shown).

CARs are synthetic, MHC-independent receptors that possess the capability to redirect immune cells such as T cells toward target cells expressing antigens.^27^ Given their ability to induce remission with otherwise-untreatable tumors, the number of CAR-T cell clinical trials has risen dramatically over the past decade.^28^ Creation of CAR-T cells by gene editing of the *TRAC* locus is particularly attractive because it allows combination of TCR knockout with high-efficiency CAR transgene integration. Prompted by the high levels of editing we observed with our systems, we aimed to test whether our nucleases are compatible with targeted CAR integration. We designed and produced high-titer AAV containing a BCMA-specific CAR linked by a 2A peptide to the tLNGFR cell-surface marker.^29,30^

The transgene was flanked by homology arms allowing for insertion of the transgene into the *TRAC* locus by homology-dependent DNA repair (HDR) by either our lead MG29-1 guide (*TRAC*-35; in exon 1) or, separately, our lead MG3-6 guide (*TRAC*-6; in exon 3) (Fig. 1C). After electroporation, T cells were recovered in culture medium containing the AAV transgene donors. We assessed knockout and knock-in efficiencies across MOIs ranging from 50,000 to 1 million vector genome copies per cell. Knock-in frequency was proportional to the AAV MOI delivered with integration exceeding 55% of cells at higher MOIs (Fig. 1D). CAR integration was robust across up to 10 T cell donors (Fig. 1E).

To validate the functionality of our CAR-T cells, we assayed cytotoxicity of the cells against K562 cells or an otherwise-isogenic K562 line expressing BCMA under a strong promoter. We purified the T cells to >95% purity using anti-tLNGFR-MACS (data not shown) and combined these T cells and target cells at different effector:target (E:T) ratios ranging from 8:1 to 0.1:1. A CAR targeting 2-nitrophenol was used as a negative control. We observed potent, BCMA-CAR-specific, antigen-specific cytotoxicity in a cell concentration–dependent manner (Fig. 1F). IFN-γ is a potent immune-activating cytokine secreted by activated CAR-T cells. We assayed IFN-γ production of our CAR-T cells compared with control T cells and observed high levels of IFN-γ secretion only when the cells were exposed to the BCMA antigen (Fig. 1G).

Finally, we expanded our edited T cells for 8 days postelectroporation, expanding them ∼100-fold from 1.6 million cells to ∼160 million cells regardless of the editing treatment (Fig. 1H). Although we have only performed this degree of cell expansion once (vs. several replicates for each of the CAR targeted integration), it indicates that gene editing with our nucleases can be compatible with the cell expansion needed to make an allogeneic CAR-T cell therapy.^25^ Altogether, we demonstrate the potential of both our type II and type V gene-editing systems to generate active CAR-T cells at high efficiency and high cell number.

### Multiplexed T cell gene editing at therapeutically relevant loci

Many clinical studies have used T cells bearing transgenic TCRs to target tumor-neoantigen-expressing cells. Simultaneous elimination of both *TRAC* and *TRBC* is important for generation of transgenic-TCR T cells as otherwise the endogenous alpha or beta chain will pair in *trans* with the transgenic TCR.^31^ Such mispairing materially reduces therapeutic TCR expression and also creates chimeric TCRs with novel and unpredictable specificities. To advance the development of safe and effective TCR-based cell therapies, simultaneous knockout of both alpha and beta chains is thus paramount. Having already shown high levels of edits at *TRAC*, we aimed to disrupt *TRBC*.

**FIG. 2.**
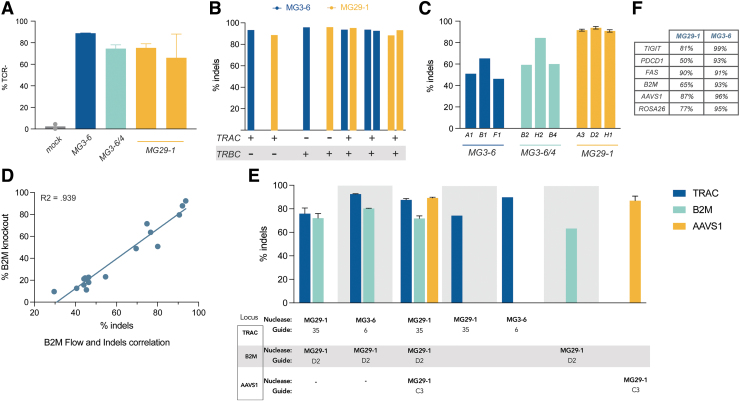
Multiplexed T cell gene editing with novel type II and type V nucleases. **(A)** Editing efficiency of top guide RNAs targeting *TRBC* found with each of three new gene-editing systems (MG3-6 in blue, MG3-6/3-4 in green, MG29-1 in yellow across two replicates); **(B)** multiplexed *TRAC* and *TRBC* gene editing with either MG3-6 or MG29-1 (MG3-6 in blue, MG29-1 in yellow). Individually formulated RNPs were delivered to T cells and gene editing assayed by DNA sequencing of *TRAC*-, *TRBC1*-, and *TRBC2*-specific amplicons. For *TRBC*, the average of the editing in *TRBC1* and *TRBC2* is reported. MG3-6 is in blue, MG29-1 in yellow; **(C)** editing efficiency of top 3 MG3-6, MG3-6/4, and MG29-1 guide RNAs targeting the *B2M* gene. Average and standard deviation across three biological replicates are given for MG29-1, representative editing results are given for MG3-6 and MG3-6/3-4; **(D)** correlation between genetic and phenotypic B2M knockout across the entire primary B2M guide screen. B2M expression of cells treated with various individual guide RNAs was measured by flow cytometry; editing was measured by amplicon sequencing; **(E)** indel analysis of *TRAC*, *B2M*, and *AAVS1* triplex gene editing. Average editing and standard deviation across two biological replicates are given. **(F)** Indel efficiency of the best performing guide RNAs from arrayed screens at the indicated loci are given for both MG3-6 and MG29-1. B2M, β2-microglobulin; RNP, ribonucleoprotein particle.

Unlike *TRAC*, targeting of *TRBC* is complicated by the fact that T cells express either one of two sequence-diverged beta-chain paralogues (*TRBC1* or *TRBC2;* despite the fact that successful beta-chain targeting is formally a duplex edit we refer to it as a single edit/knockout throughout this article as the gene editing results in inactivation of only one protein). We designed guide RNAs for MG3-6, MG29-1, and a third nuclease, MG3-6/3-4, targeting both *TRBC1* and *TRBC2* and then assayed TCR expression by flow cytometry 3 days postelectroporation. We observed a spectrum of TCR reduction frequencies: samples with intermediate TCR reduction correlated with guide RNAs predicted to target either *TRBC1* or *TRBC2*, whereas the guides with a profound reduction in TCR expression were those that are predicted to target both *TRBC1* and *TRBC2*, the best four of which are given (Fig. 2A).

To make duplex *TRAC*/*TRBC* knockout cells we cotransfected mixtures of independently assembled RNPs targeting both *TRAC* and *TRBC*. It is not possible to monitor duplex editing via flow cytometry as inactivation of either *TRAC* or *TRBC* is sufficient to generate a TCR-negative T cell. Instead, we measured editing at the DNA level by amplicon sequencing, developing a nested PCR assay for *TRBC*, whereby the primers in the first amplification step target sequences discordant between *TRBC1* and *TRBC2* followed by a second amplification using guide-specific primers. When gene editing was thus assayed, we found that duplex *TRAC* and *TRBC* editing took place at extremely high frequencies with no decrement whatsoever to the efficiencies seen with individual-locus editing (Fig. 2B). Of note, duplex editing worked well using MG3-6, MG29-1, and also with both possible combinations of MG3-6 and MG29-1.

Ablation of the TCR prevents therapeutic cells from attacking their host. Reciprocally, effective allogeneic cell therapy in immuno-oncology requires that the body does not attack the transplanted cells, that is, that the cells persist in the recipient. Adoptively transferred T cells will display endogenous peptides in class I HLA receptors. Natural human genetic variation within such peptides will result in donor cells being perceived as foreign. Removal of β2-microglobulin (B2M), a structural component of all class I HLA complexes, effectively prevents foreign cell antigen display and rejection of donor cells by the adaptive immune system. We therefore used our novel gene-editing tools to target the *B2M* gene, finding many guide RNAs that produce indels at a high frequency (Fig. 2C). We further characterized our lead MG29-1 guides by assessing editing at different doses and by parallel flow cytometry and NGS experiments, observing a clear, dose-dependent correlation between the two assays (Fig. 2D).

A major advantage of *ex vivo* cell therapy is the facility of engineering multiple edits into the genome. Having demonstrated high levels of single and duplex editing at three therapeutically relevant targets in T cells, we next attempted multiplexed editing with our nucleases. To test the efficacy of our systems to generate such complex gene edits, we cotransfected independently assembled RNPs targeting *TRAC*, *B2M*, and *AAVS1* with either MG3-6 and/or MG29-1 and assayed knockout by NGS. Analysis of the genomic DNA revealed that up to 88%, 73%, and 90% of the cells bore edits at *TRAC*, *B2M*, and *AAVS1*, respectively (Fig. 2E). As with our duplex TCR knockout, these high levels of editing occurred both within and across editing systems at *TRAC and B2M*, expanding the potential range of applications for complex edits enabled by our enzymes.

To further explore the scope of our gene-editing systems, we also performed guide screens with MG3-6 and MG29-1 targeting additional genes advantageous for cell-based therapies, including *TIGIT*, *FAS*, and *PDCD-1* (PD-1). The efficiencies of our most active guides targeting these genes is given in Figure 2F. In all cases, we were able to find potent guides with both of our systems.

### NK cell engineering with novel gene-editing nucleases

Although traditional cellular immunotherapy strategies are dominated by T cell–expressed CARs, NK cells have more recently gained traction as an alternative cell therapy platform. NK cells possess a cytotoxic potential similar to T cells but unlike T cells, NK cells intrinsically lack alloreactivity and do not trigger side effects common to CAR-T administration such as cytokine release syndrome and immune effector cell–associated neurotoxicity syndrome.^32–34^ Encouraged by the results of our T cell experiments, we sought to perform analogous experiments in NK cells. To demonstrate the receptivity of NK cells to gene editing with our nucleases, we targeted the *CD38* gene, a cell surface receptor responsible for NK cell fratricide in response to daratumumab infusion.^35,36^ We expanded purified primary NK cells and transfected them with a high-efficiency electroporation protocol.^37^

**FIG. 3.**
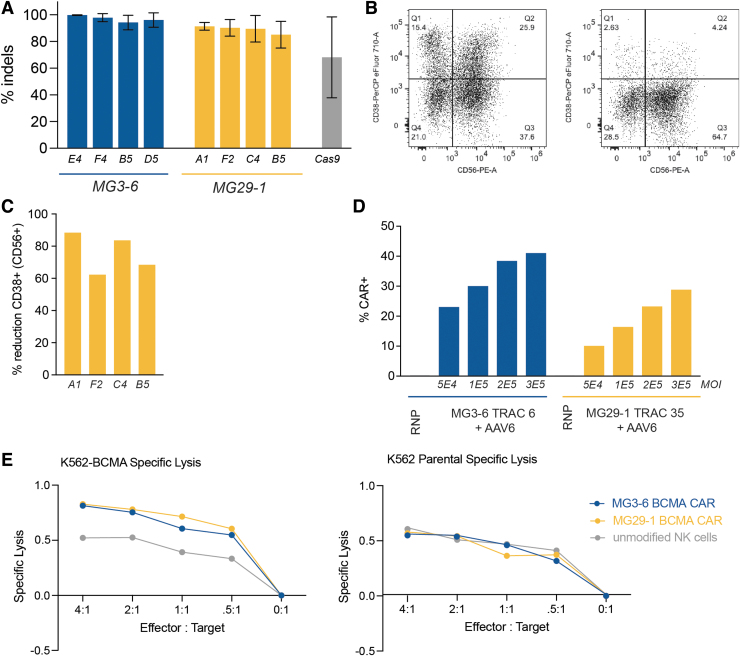
NK cell engineering with novel gene-editing nucleases. **(A)** Average indel frequencies of top 4 CD38 guides for MG3-6 and MG29-1 are given in blue and yellow, respectively. Error bars indicate standard deviations across three replicates; **(B)** flow cytometry analysis of NK cell populations before (left) and after (right) gene editing with MG29-1 guide A1. **(C)** Percent reduction of CD38^+^ cells within the CD56^+^ NK cell population with top 4 MG29-1 guides. Knockout was assayed by flow cytometry across two biological replicates. **(D)** Dose response of CAR integration and expression at the TRAC locus in NK cells as a function of AAV genomes transduced with either MG3-6 (blue) or MG29-1 (yellow) RNP; **(E)** target-cell lysis by MG-nuclease-generated CAR NK cells. Lysis of either K562 cells (left) or K562-BCMA cells by unmodified NK cells (gray), MG3-6-edited NK cells (blue), or MG29-1-edited NK cells (yellow). NK, natural killer.

We assayed *CD38* knockout efficiency 5 days postnucleofection, finding several highly active guide RNAs for both MG3-6 and MG29-1 (Fig. 3A). We further validated the editing of lead guides at two different doses and observed comparable levels of editing at both doses (data not shown). We performed a parallel analysis by flow cytometry, finding that we could eliminate CD38 expression in >80% of CD38^+^ cells (Fig. 3B, C). Using the *TRAC*-targeting reagents from our T cell experiments, we created CAR-NK cells by integrating the BCMA-CAR into the NK cell genome (Fig. 3D). Despite NK cells' high intrinsic reactivity to the K562 cell line,^38^ we saw a CAR- and BCMA-dependent NK cell cytotoxic response (Fig. 3E).

### Novel type II and type V nucleases are highly active across a broad panel of primary cells

**FIG. 4.**
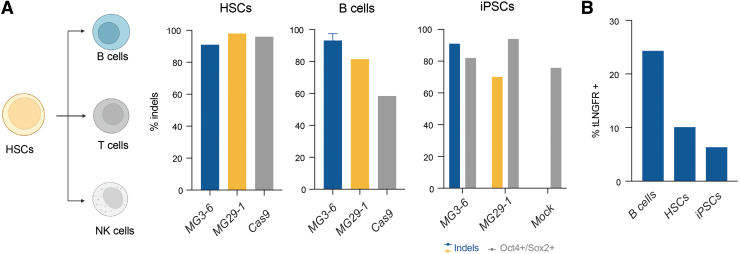
Broad primary cell gene editing with novel nucleases. **(A)** Editing of human HSCs (left), human primary B cells (middle), and iPSCs (right) at *TRAC* with MG3-6 (blue), MG29-1 (yellow), and Cas9 (gray) across two biological replicates. For iPSCs, the percentage of cells that are Oct4^+^ and Sox2^+^ is also shown, in gray; **(B)** targeted integration frequencies at TRAC from B cells, HSC, and iPSCs as measured by flow cytometry for the tLNGFR marker. HSC, hematopoietic stem cell; iPSC, induced pluripotent stem cell.

Inspired by our successful gene editing results in T and NK cells, we sought to broaden the repertoire of primary cells in which our nucleases are effective. More specifically, gene editing in iPSCs, HSCs, and other hematopoietic-lineage immune cells, such as B cells present attractive and powerful targets for the development of cell therapies. To that end, we applied our lead MG3-6 and MG29-1 *TRAC* reagents to effect gene editing in these three cell types. As with our T cell and NK cell work, we observed >90% indel formation in B cells, HSCs, and iPSCs with MG3-6 and MG29-1 (Fig. 4A). We next tested the ability of our enzymes to stimulate HDR in these cell types. Using the AAV from our T and NK cell experiments, we demonstrate AAV-6-mediated transgene integration and expression (Fig. 4B).

### Gene-editing capabilities of MG3-6, MG3-6/4, and MG29-1

**FIG. 5.**
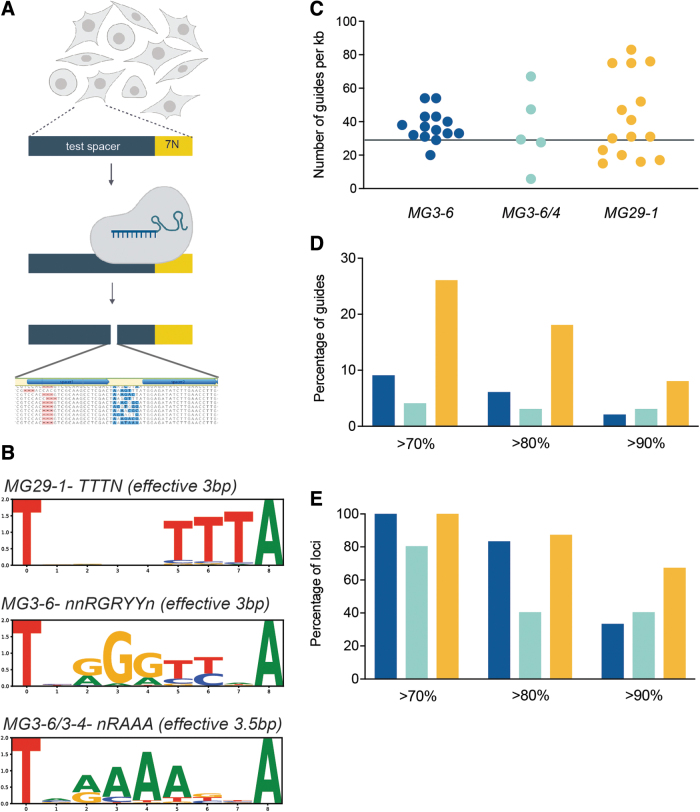
Gene editing capabilities with MG3-6, MG3-6/4, and MG29-1. **(A)** Schematic of in-cell, library-based assay for determining nuclease PAM sequences. **(B)** Representative seqLogo PAM sequences for MG29-1, MG3-6, and MG3-6/4 as determined from the in-cell assay. The first and last bases of the candidate PAM sequences are fixed as T and A, respectively. Consensus PAMs and effective length are shown above SeqLogo. **(C)** Targeting density (number of guides/kb DNA) of the three nucleases across 4–15 targets. MG3-6 is shown in blue, MG3-6/4 in green, and MG29-1 in yellow. Horizontal line represents expected target density for a nuclease with a 3-bp PAM (31.25 guides/kb); **(D)** percentage of all screened guides that result in indel formation at or above the indicated frequency (70%, 80%, or 90%). The color scheme is as in **(C)**; **(E)** percentages of all loci targeted for which indels were able to be generated at or above the indicated frequency (70%, 80%, or 90%). The color scheme is as in **(C, D)**. PAM, protospacer-adjacent motif.

Gene-editing system flexibility is measured by the targeting density, that is, the probability that a DNA sequence of average base composition can be targeted. For CRISPR-associated systems, targeting density is dependent on the frequency of the PAM sequence. To determine the PAM sequences of our enzymes we used an initial *in vitro* plasmid-based library approach.^18,19^ As this method can broaden the specificity of the PAM sequence relative to that found in cells, we next used a screen in human cells using a lentiviral-based library (Fig. 5A).^39^ Inspection of cleaved library members allowed us to infer the following consensus PAM sequences for our enzymes: 5′-NNRGRYY-3′ (MG3-6), 5′-NNRAAW-3′ (MG3-6/4), and 5′-TTTN-3′ (MG29-1) (Fig. 5B). These PAMs are also supported by inference from cumulative editing data from guide screening approaches using the broader, *in vitro*-derived PAMs (data not shown).

Although both of our type II enzymes' PAM sequences are more than three DNA base pairs, they tolerate degeneracy in such a way as to make the PAM frequency equivalent to the 3 base pair PAM of MG29-1. Any 3 base pair sequence will occur once every 64 base pairs in DNA of random sequence composition. As both strands are targetable, the theoretical targeting density for each enzyme is therefore once every 32 base pairs or ∼31 times per kilobase of DNA. Figure 5C provides the actual PAM frequencies for MG3-6 and MG29-1 across many targeted loci in the human and mouse genomes. Across ∼31 kb of target genomic DNA we found roughly 40 guides/kilobase for each system with a wide variance by locus.

Gene-editing system utility is a function of both targeting density as well as the fraction of guide RNAs that are highly active. With >1000 guide RNAs tested in primary cells, we find that 10% of MG3-6 guide RNAs and fully 25% of MG29-1 guide RNAs induce indels in >70% of chromosomes. Furthermore, ∼10% of all MG29-1 guide RNAs create indels at a >90% frequency (Fig. 5D). This global frequency of highly active guide RNAs is not owing to a concentration of such guide RNAs within a few, highly targetable loci. Rather, highly active guides are dispersed broadly across the targets and thus contribute to a high overall project success rate: at all loci tested there is at least one guide that can edit >70% of chromosomes for MG3-6 and for MG29-1 (Fig. 5E). More than two-thirds of target loci have an MG29-1 guide RNA that creates indels in >90% of chromosomes (Fig. 5E). Cumulatively (considering editing by any nuclease) all targets had a guide RNA that could create indels 80% of the time and >95% of targets could be edited with 90% efficiency.

### MG3-6 and MG29-1 can be highly specific nucleases

Although it is critical for gene-editing systems to have high activity, high specificity is equally important. Indeed, Cas9 is notorious for tolerating multiple PAM-distal mismatches and thus cleaving multiple sites in the genome. To assay the genome-wide specificity of our systems we used a technique that uses the spontaneous capture of an oligonucleotide in HEK293T cells to mark the site of DNA double-strand breaks (DSBs). We analyzed the DSB spectra of two well-characterized Cas9 guide RNAs, one with a very poor specificity profile (VEGF-A) and another that cleaves at only two to three sites (*TRAC*) as well as a variety of highly active guides for MG3-6 and MG29-1.

**FIG. 6.**
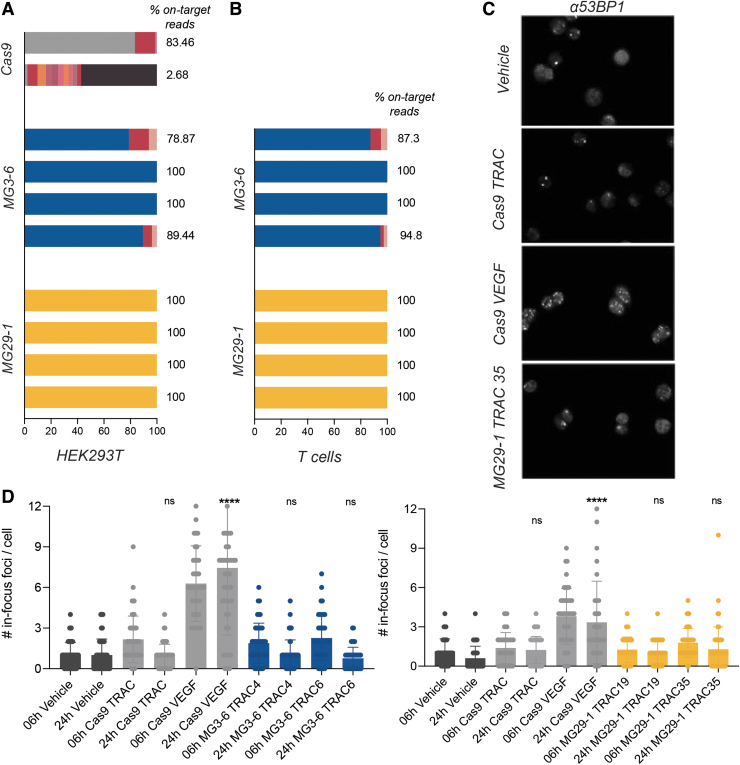
Gene-editing specificity of novel nucleases. **(A)** DSB discovery via capture of a double-stranded oligonucleotide in HEK293T cells with Cas9 (on target read in gray), guides MG3-6 TRAC B2, TRAC D2, TRAC 6, and GR 3 (on target in blue), and MG29-1 guides TRAC 9, TRAC 19, TRAC 35, and GR 13 (on target in yellow) across three biological replicates. **(B)** DSB discovery via capture of a double-stranded oligonucleotide in primary T cells using the same MG3-6 and MG29-1 guides from **(A;** averaged across three biological replicates**)**; **(C)** representative immunofluorescence images obtained from costaining nuclease-treated HEK293T cells with DAPI and an antibody that detects the DNA repair protein 53BP-1; **(D)** quantitative analysis of 53BP-1 foci formation either 6 or 24 h after transfection with RNPs targeting the indicated loci (*N* = 37–69, mean and standard deviations are shown. ****p < 0.0001 as determined by an unpaired Student's *t*-test; ns, not significant). DSB, double-strand break.

For Cas9, we recapitulated the published list of off-target sites with the VEGF-A guide, finding 23 of the top 24 previously identified off-target sites, with 87% of our total sequence reads residing in loci shared with the published list, and with the detection of an additional 113 off-target sites (despite the assay being done in a different cell type).^40^ We conclude that our implementation of this assay is equivalent to that found in the literature. Considering guides for MG3-6 and MG29-1, the majority of our MG3-6 guides and all our MG29-1 guides showed DSB formation only at the intended target site, even at RNP doses that saturate on-target editing (Fig. 6A). When repeated in primary T cells, the frequency of off-target gene-editing sites decreased compared with when assayed in HEK293T cells (Fig. 6B).

To validate this observation, we developed a molecularly orthogonal nuclease specificity assay based on the induction of DNA repair foci. The 53BP1 protein is an essential component of the DNA repair signaling cascade in mammalian cells, forming a punctum (or focus) at sites of DSBs and persisting for at least 4 h once established.^40^ As efficient gene editing in diploid cells should induce two DSBs per cell, two 53BP1 foci should appear during the editing process and should dissipate thereafter. We quantified the number and duration of 53BP1 puncta induced by the Cas9 guides used in the DSB discovery assay described previously as well as several MG3-6 and MG29-1 guide RNAs.

For the poorly specific *VEGF-A* Cas9 guide RNA we observed a marked increase in the number of 53BP1 puncta; for the (relatively) specific *TRAC* Cas9 guide RNA only the expected increase in foci was seen (Fig. 6C). The number of 53BP1 foci induced by the Cas9 *TRAC* guide RNA returned to baseline when assayed 24 h post-transfection. Conversely, the number of puncta induced by the *VEGF-A* guide remained high at this time point (Fig. 6D). When MG3-6 and MG29-1 gene-editing reagents were assayed, we observed that the average number and duration of 53BP1 foci was comparable with that observed with the relatively specific Cas9 guide. We conclude from this assay that gene editing with MG3-6 and MG29-1 do not cause grossly unacceptable off-target gene editing. Taken together with the DSB discovery assay, the evidence suggests that our lead gene-editing systems have a very favorable degree of specificity.

### Novel type II and type V nucleases lack preexisting antibody or T cell immune response

Activity and specificity are the *sine quibus non* of gene editing. However, to create an effective human therapeutic it is important that prospective patients be receptive to treatment. *S. pyogenes* and *Staphylococcus aureus* are common human pathogens; a recent study demonstrated a high incidence (30–50%) of preexisting immunity to Cas9.^16^ The consequences of preexisting immunity will depend on the gene-editing delivery mode: preexisting Cas9 antibodies will clear serum-exposed RNPs; a parallel memory T cell–based immunity will put edited cells at risk for rapid cytotoxic attack, particularly in situations where the gene-editing reagents (or nucleic acid encoding them) are the drug substance.^41,42^ Whereas MG3-6 is sourced from the human microbiome, MG29-1 was discovered in a sample from a hydrothermal vent. We postulated that because our nucleases are not known to be from human pathogens that they would be immuno-naive.

**FIG. 7.**
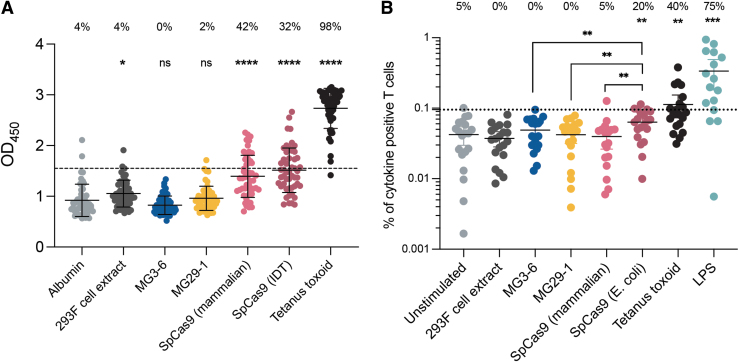
Assaying preexisting antibody or T cell immune response to the novel nucleases. **(A)** Detection of antibodies against MG-3-6 and MG29-1 (*N* = 48) by ELISA with 1:50 serum dilutions. Tetanus toxoid was used as the positive control. Serum samples above the dashed line (mean absorbance of the negative control [human albumin] plus two standard deviations from the mean) were classified as antibody positive. *p < 0.05, ****p < 0.0001 as determined by an unpaired Student's *t*-test; ns, not significant. The percentage values above each group indicate the percentage of donors that were positive for an antibody response to each different antigen. **(B)** Nuclease-specific T cell responses in humans. Frequency of T cells that was positive for a cytokine in response to stimulation with each different antigen. Data points above the dotted line are considered positive for a cytokine response. The threshold was determined by calculating the mean frequency of cytokine-positive T cells in the unstimulated group and adding two times the standard deviation of the mean. The percentage values above each group indicate the percentage of donors that were positive for a cytokine response to each different antigen. The error bars indicate the mean and 95% CI. **p < 0.01, ***p < 0.001, paired *t*-test; unless specified, each dataset was tested against the unstimulated control for significance (*n* = 20; *n* = 16 for LPS control group). CI, confidence interval; ELISA, enzyme-linked immunosorbent assay; LPS, lipopolysaccharides.

We probed human sera from 48 donors for the presence of antibodies against our nucleases and Cas9 using an ELISA-based assay.^16^ To eliminate the possibility of a positive result stemming from antibodies to a contaminating *E. coli* protein and to prevent interference from endotoxin we used HEK293 cells to express MG3-6, MG29-1, and Cas9 rather than *E. coli*. Almost all samples lacked immunoreactivity to human albumin and to a mock-purified 293 cell preparation; almost all samples were reactive to tetanus toxoid (Fig. 7A). We confirmed the previously described preexisting immunity to Cas9 with both the bacterial- (42%) and mammalian-produced (32%) lots of Cas9 (Fig. 7A). In contrast, we observed no significant response to either MG3-6 or MG29-1.

We next probed for corresponding T cell–based immunoreactivity. PBMCs were incubated with the nucleases and the T cell subset subsequently probed for production of three cytokines induced by T cell activation, IL-2, TNF-α, and IFN-γ. In this system, B cells in the PBMC mixture take up the protein and serve as antigen-presenting cells for the commingled T cells. Tetanus toxoid and endotoxin were used as positive controls. Commercially produced Cas9 elicited a modest but significant increase in the frequency of immunoreactive cells. By contrast neither MG3-6 nor MG29-1 induced appreciable cytokine production (Fig. 7B).

## Discussion

In this study, we demonstrate the characterization of our MG3-6, MG3-6/4, and MG29-1 RNA-directed nucleases and their application to a wide variety of focus areas in cell-based therapy. Our results with MG29-1 are noteworthy as in one enzyme there are three significant technical advantages over competing gene-editing nucleases: (i) MG29-1 has gene-editing activity roughly equal to that of Cas9; (ii) MG29-1 possesses the intrinsically improved specificity typical of type V enzymes; and (iii) MG29-1 lacks detectable preexisting immunoreactivity. Overall, we believe MG29-1 in particular could be a best-in-class gene-editing nuclease.

Overall, the ability of our nucleases to effect targeted CAR integration into *TRAC* is quite similar to that seen with Cas9.^20^ We saw a lower frequency of transgene integration with MG29-1 compared with MG3-6 (Fig. 1E). We suspect this deficit stems from slightly suboptimal homology arm positioning in the transgene donor construct: whereas the propinquity of the MG3-6 DSB and the homology arm ends ensure maximal usage of the transgene donor, the MG29-1 donor homology arms are offset by several base pairs from the DSB, perhaps just enough for the 3′ end to use the unintended homology arm of the donor for DNA repair (and in doing so, fail to copy the transgene sequence into the chromosome).

Furthermore, and also unlike type II enzymes, this positioning of the homology arm end outside the guide RNA sequence ensures that the nuclease target site is recreated in the repaired allele. Subsequent recleavage of the CAR-bearing allele might in some cases cause loss of CAR expression. Once integrated however, we find no difference in CAR biology between MG29-1- and MG3-6-edited cells despite the CAR being inserted into different exons of *TRAC* (exons 1 and 3, respectively).

Our three gene-editing nucleases each have PAM sequences expected to occur once every 32 base pairs in DNA. Considered individually, each is fourfold more restrictive than Cas9's 2 base pair PAM. Considered collectively however, the targeting density of the current Metagenomi editing platform is approximately once every 16 base pairs. (The PAM sequences of MG29-1 and MG3-6/4 are close to the reverse complement of each other and, therefore, conservatively, are not additive.) As additional nucleases are added to our gene-editing platform, the design density will grow proportionately.

Our in-cell gene-editing specificity assay reported a relative increase in specificity when performed in primary T cells as opposed to immortalized HEK293T cells. Idiosyncratic, cell-specific differences in chromatin accessibility are known to affect the frequency of gene editing even when the cell types in question are dividing rapidly and we suspect this underlies our observation here.^43–48^

Monitoring the process of DNA repair in cells using 53BP-1 immunofluorescence has a long history in gene-editing research.^45–47^ However, as this technique is necessarily destructive to the cell, it can afford only a snapshot of on-going DNA repair and is thus intrinsically less sensitive than methods that mark sites of DSBs for later cataloging. However, the lowered sensitivity for this temporal reason is balanced by the technique's likely ability to detect the great majority of DSBs under repair. By contrast, capture of a tagging molecule at the DSB typically occurs <10% of the time.^49^

Our investigation of preexisting immunity to our nucleases recapitulated Charlesworth et al.'s work with Cas9 regarding antibody-based immunity, whereas we found a real-but-more-modest degree of T cell preexisting immunity to Cas9 purified from *E. coli* and no T cell response to Cas9 purified from 293T cells. The reason for this difference is unclear but we do note that in general we find an ∼10-fold higher background frequency of cytokine-expressing cells than in the published work. Such an effect would serve to compress any difference with true cytokine-positive cells.

In sum, we establish three new CRISPR-associated enzymes as high-efficiency gene-editing tools capable of achieving a variety of genetic engineering outcomes pertinent to cell therapy development. We look forward to their use to make new cell-based medicines and to their translation to gene editing in the body itself.
